# NOD1 modulates IL-10 signalling in human dendritic cells

**DOI:** 10.1038/s41598-017-00691-x

**Published:** 2017-04-21

**Authors:** Theresa Neuper, Kornelia Ellwanger, Harald Schwarz, Thomas A. Kufer, Albert Duschl, Jutta Horejs-Hoeck

**Affiliations:** 1 Department of Molecular Biology, University of Salzburg, Salzburg, Austria; 2 Institute of Nutritional Medicine, Department of Immunology, University of Hohenheim, Stuttgart, Germany

## Abstract

NOD1 belongs to the family of NOD-like receptors, which is a group of well-characterised, cytosolic pattern-recognition receptors. The best-studied function of NOD-like receptors is their role in generating immediate pro-inflammatory and antimicrobial responses by detecting specific bacterial peptidoglycans or by responding to cellular stress and danger-associated molecules. The present study describes a regulatory, peptidoglycan-independent function of NOD1 in anti-inflammatory immune responses. We report that, in human dendritic cells, NOD1 balances IL-10-induced STAT1 and STAT3 activation by a SOCS2-dependent mechanism, thereby suppressing the tolerogenic dendritic cell phenotype. Based on these findings, we propose that NOD1 contributes to inflammation not only by promoting pro-inflammatory processes, but also by suppressing anti-inflammatory pathways.

## Introduction

NOD-like receptors (NLRs) comprise a family of cytosolic pattern recognition receptors (PRRs) that serves as a first line of defence against invading pathogens. The human NLR family contains 22 members that are structurally conserved. NLRs possess a leucine-rich repeat (LRR) domain for ligand sensing, a NOD domain that facilitates self-oligomerisation upon activation, and an effector domain that mediates downstream signalling. NOD1 and NOD2, the two best-characterised NLRs, recognise specific fragments of bacterial peptidoglycan^1, 2^. Ligand sensing leads to NF-κB activation and mitogen-activated protein kinase (MAPK)-signalling which results in the production of pro-inflammatory cytokines and chemokines^3–6^.

Recently, peptidoglycan-independent functions of NOD1 and NOD2 have been described. For example, it was reported that NOD1 senses the activation status of small Rho GTPases^7^, and activation of NOD1/2 was linked to ER stress responses^8^. Moreover, low levels of NOD1 have been correlated with enhanced tumour growth of the breast cancer cell line MCF-7, and NOD1 expression was diminished in biopsies derived from gastric carcinoma patients^9, 10^. In line with this, *in vivo* studies utilising a model of colitis-associated colon tumourigenesis revealed that NOD1-deficient mice are prone to develop inflammation-induced tumours. In the latter, transfer of wild-type bone marrow was sufficient to rescue NOD1-deficient mice from developing tumours^11, 12^. This indicates that NOD1 may have tumour suppressive functions which are at least partially mediated by cells of the haematopoietic compartment.

Amongst the cells derived from haematopoietic progenitors, dendritic cells (DCs) are key players in maintaining the balance between immunity and tolerance. Whereas effector T-cell responses are initiated by mature DCs that have been primed by particular pathogen-associated factors^13^, regulatory T (Treg) cell responses are mainly induced by immature (iDCs)^14, 15^ or tolerogenic DCs^16–18^. Tolerogenic DCs occur *in vivo* as specific DC subsets^18–22^, and they can also be differentiated *in vitro* by various protocols^23^. In malignancies, tumour- or stroma-derived factors usually promote the differentiation and accumulation of tolerogenic DCs, thus blocking anti-tumour immunity^24, 25^. Both naturally occurring and induced tolerogenic DCs are characterised by low expression of interleukin (IL)-12 and high IL-10 secretion^26^. IL-10 can convert iDCs into tolerogenic DCs that secrete IL-10 and further stimulate the generation of Treg cells^16, 18, 26^. IL-10 exerts its functions by receptor-mediated activation of JAK kinases, resulting in the recruitment and phosphorylation of the transcription factors STAT1 and STAT3 and the subsequent activation of target gene expression^27–29^. Signalling induced by cytokines, including IL-10, is regulated by a family of intracellular suppressor of cytokine signalling (SOCS) proteins. The eight members of the SOCS family (CIS and SOCS1–SOCS7) are important inhibitors of JAK/STAT activation and play key roles in regulating innate and adaptive immune responses^30^.

Recent findings linking NOD1 to functions independent of peptidoglycan-sensing mainly concern pro-inflammatory processes. A potential role for NOD1 in anti-inflammatory signalling has not yet been extensively investigated. Here we demonstrate that, in human DCs, decreased NOD1 levels promote a tolerogenic DC phenotype and alter IL-10-induced STAT signalling by a mechanism involving SOCS2.

## Results

### NOD1 silencing enhances IL-10-dependent *il10* and *march1* expression and inhibits CD86 and IL-12 levels

**Figure 1:**
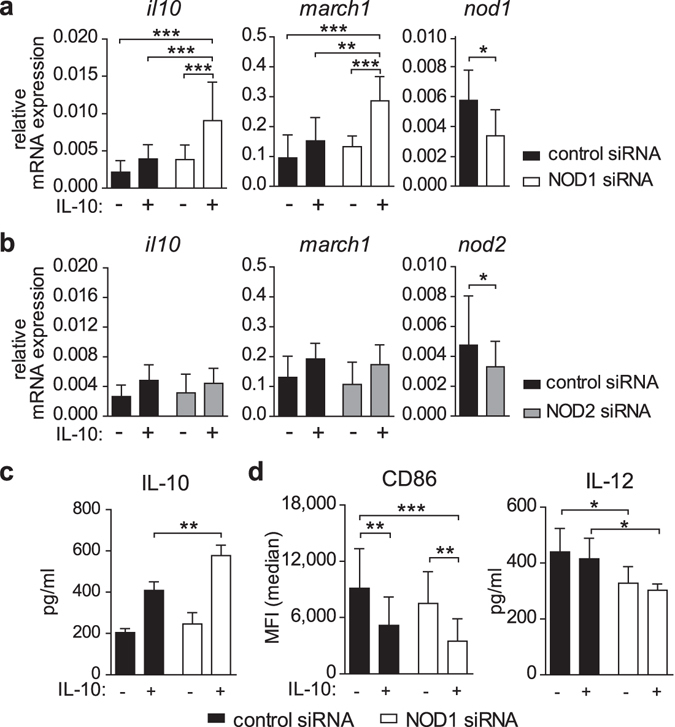
NOD1 silencing promotes *il10* and *march1* expression and decreases CD86 and IL-12 levels. (**a/b**) Immature DCs were generated and transfected with a control siRNA or siRNA directed against NOD1 (**a**) or NOD2 (**b**). 72 hours post transfection, cells were analysed for silencing efficiency or stimulated with IL-10 (30 ng/ml), and IL-10 target gene expression was monitored 2 hours post IL-10 induction. Data represent mean and SD of at least four independent experiments. (**c**) After 3 days of control or NOD1 silencing, DCs were induced with IL-10 for 4 hours. Cells were re-plated and cultured in fresh medium without IL-10 for 48 hours. IL-10 secretion was analysed after 48 hours by ELISA. Data represent mean and SD of four independent experiments. (**d**) 48 hours post IL-10 stimulation, CD86 expression and IL-12 secretion of control or NOD1 silenced DCs were evaluated by flow cytometry and ELISA, respectively. Data represent mean and SD of at least five independent experiments. For statistical analysis a one-way ANOVA with Tukey’s post hoc test was performed.

IL-10 is a potent anti-inflammatory cytokine that exerts its function on various target cells, including DCs. IL-10-induced tolerogenic DCs are characterised by (i) elevated *il10* and *march1* gene expression, (ii) weak up-regulation of co-stimulatory molecules, and (iii) enhanced ability to promote Treg generation^18, 31^. To investigate whether NOD1 activation affects IL-10 functions in iDCs, we stimulated iDCs with both IL-10 and a NOD1 ligand, γ-D-glutamyl-meso-diaminopimelic acid (iE-DAP), and analysed IL-10-dependent gene expression. To verify NOD1 activation by iE-DAP stimulation in iDCs, we monitored *tnfα* mRNA levels. Stimulation of iDCs with iE-DAP resulted in enhanced *tnfα* expression, which was significantly decreased by the presence of IL-10 (Supplementary Fig. S1). In contrast, upregulation of the IL-10 target genes *il10* and *march1* was not altered in the presence of iE-DAP (Supplementary Fig. S1). Because NOD1 activation by its ligand did not affect the IL-10 target genes examined, we next analysed whether the depletion of endogenous NOD1 has an effect on IL-10-induced signals. Therefore, we treated human iDCs with NOD1 siRNA three days prior to IL-10 treatment and analysed the expression of IL-10 targets by q-RT-PCR and flow cytometry. To exclude immunologically relevant side effects due to siRNA treatment, we monitored type I interferon responses. The observed lack of interferon α expression suggests that the siRNA treatment did not activate antiviral PRRs (Supplementary Fig. S2). To control for the specificity of NOD1-dependent effects, we additionally used NOD2-siRNA-treated iDCs or iDCs transfected with a control siRNA. We found that IL-10-induced cells, in which NOD1-expression was targeted, expressed significantly higher amounts of *il10* and *march1* (Fig. 1a), whereas reduction of NOD2 had no effect on target gene expression (Fig. 1b). To analyse the effects of NOD1 depletion in iDCs in more detail, we quantified IL-10 secretion and also monitored the induction of the co-stimulatory molecule CD86 and the pro-inflammatory cytokine IL-12 two days post IL-10 treatment. NOD1 silencing of IL-10 treated iDCs results in elevated IL-10 protein levels compared to control silenced cells (Fig. 1c), whereas both CD86 and IL-12 levels were slightly decreased (Fig. 1d). The use of additional siRNAs targeting NOD1 confirmed that NOD1 silencing efficiency was directly correlated to the induction of IL-10 mRNA expression (Supplementary Fig. S3).

### Reduced NOD1 expression supports a tolerogenic DC phenotype

**Figure 2:**
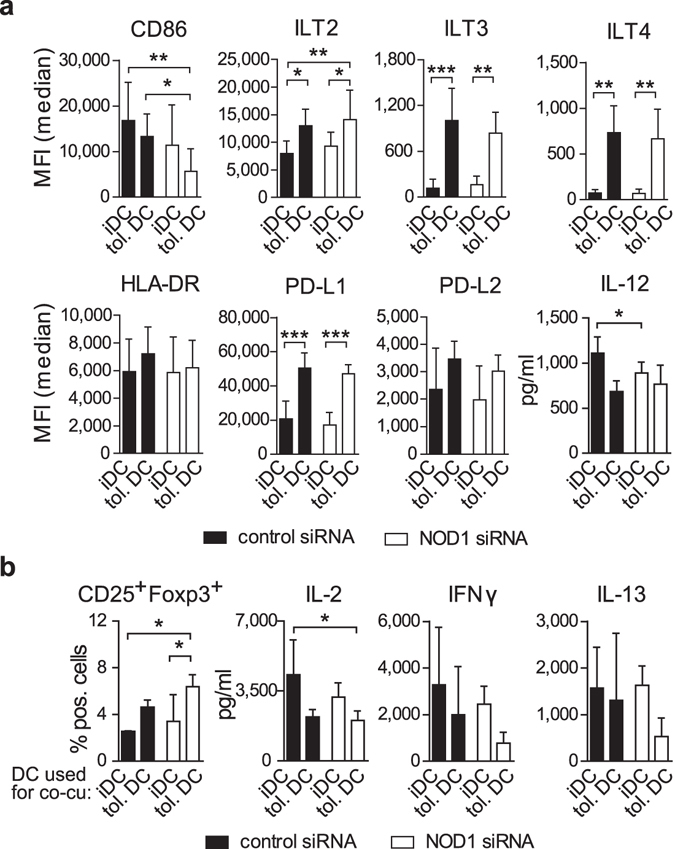
NOD1 silencing promotes a tolerogenic DC phenotype. (**a**) To obtain tolerogenic DCs (tol. DC), iDCs were differentiated in the presence of IL-10 (30 ng/ml) for 7 days. Fully differentiated tolerogenic DCs and conventional iDCs (differentiated in the absence of IL-10) were silenced with control or NOD1 siRNA for 72 hours. ILT2, ILT3, ILT4, HLA-DR, CD86, PD-L1 and PD-L2 were analysed by flow cytometry. IL-12 secretion was monitored by ELISA. Data represent mean and SD of at least three independent experiments. (**b**) iDCs or tolerogenic DCs were co-cultured (co-cu) with naïve, allogeneic CD4^+^ T-cells. After 12 days of co-culture, only T-cells were re-stimulated with anti-CD3/PMA for 48 hours, and CD25 and FOXP3 expression was analysed by flow cytometry, and IL-2, IFN-γ and IL-13 secretion was measured by multiplex assay. Lymphocytes were gated on live, CD3^+^, CD4^+^ cells after doublet exclusion. Data represent mean and SD of three independent experiments. For statistical analysis a one-way ANOVA with Tukey’s post hoc test was performed.

As described previously, IL-10 treatment of iDCs for 7 days leads to the generation of a tolerogenic DC phenotype that is characterised by high levels of IL-10, but low IL-12 secretion^18^. Moreover tolerogenic DCs express high levels of the Ig-like transcripts ILT2, ILT3, and ILT4 which act as inhibitory molecules to prevent DC maturation^18^. To further investigate the consequences of NOD1 depletion on the function of tolerogenic DCs, iDCs were differentiated in the presence of IL-10 for 7 days to obtain tolerogenic DCs, and NOD1 silencing was performed on day 5. Compared to iDCs, tolerogenic DCs showed higher levels of ILT2, ILT3, ILT4, HLA-DR and PD-L1/2, two additional surface molecule that have been shown to provide inhibitory signals^32^, whereas there was no difference between control and NOD1-silenced tolerogenic DCs. However, NOD1-silencing led to significant down-regulation of IL-12 secretion in iDCs and diminished the expression of the co-stimulatory surface marker CD86 in tolerogenic DCs and iDCs (Fig. 2a).

To determine whether NOD1 silencing in tolerogenic DCs also modulates their capacity to induce T-cell responses, we performed co-culture experiments with allogeneic, naïve CD4^+^ T cells. After 12 days, we harvested the T cells and re-stimulated them with α-CD3 and phorbol ester (PMA) for an additional 48 hours. The percentage of CD4^+^/CD25^+^/FOXP3^+^ T cells was enhanced upon co-culture with NOD1-silenced tolerogenic DCs compared to control-siRNA-treated cells (Fig. 2b). Analysis of T cell-derived cytokines confirmed that T cells co-cultured with NOD1-silenced tolerogenic DCs released lower amounts of T cell cytokines IL-2, IFN-γ and IL-13 compared to T cells that were co-cultured with control tolerogenic DCs.

Taken together, our data show that NOD1 silencing supports a tolerogenic, anti-inflammatory DC phenotype by enhancing *il10* and *march1* gene expression as well as by down-regulating CD86 and IL-12 production, resulting in better priming capacities to generate regulatory T cells.

### Reduced NOD1 expression dampens IL-10-induced STAT1 phosphorylation but increases STAT3-DNA binding

**Figure 3:**
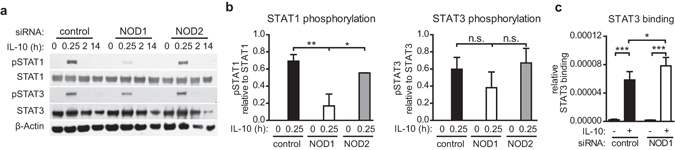
Decreased STAT1 activation, but enhanced STAT3 DNA-binding, upon NOD1 silencing. (**a/b**) Immature DCs were generated and transfected with a control siRNA or siRNA directed against NOD1 or NOD2. STAT activation was analysed by Western Blot. Transfected iDCs were stimulated with IL-10 for the indicated time points and tyrosine phosphorylation of STAT1 and STAT3 was detected by specific antibodies. To control for equal loading, total STAT protein and β-actin were detected. One of two independent experiments (**a**) and quantification of two experiments (**b**) are shown. Data represent mean and SD. (**c**) IL-10 (30 ng/ml)-induced STAT3 binding was analysed after 60 minutes by means of a STAT3 binding assay. STAT3 binding was normalised to total STAT3 protein in the cytosolic fraction, which was measured by Western Blot analysis. Data represent mean and SD of three independent experiments. For statistical analysis a one-way ANOVA with Tukey’s post hoc test was performed.

We next analysed the effect of NOD1 on the IL-10 signalling cascade in more detail. Binding of IL-10 to its receptor induces the activation of JAK kinases, resulting in the recruitment and phosphorylation of STAT1 and STAT3 and subsequent binding of these transcription factors to promoter regions of IL-10 target genes^27–29^. To investigate whether the observed increase in *il10* expression (Fig. 1a) resulted from enhanced activation of STAT proteins, we examined IL-10-induced phosphorylation of STAT1 and STAT3 in iDCs transfected with control, NOD1 or NOD2 siRNA. We found that NOD1-silencing leads to a significant reduction of IL-10-induced STAT1 phosphorylation (pY701) compared to control cells (Fig. 3a,b), whereas STAT3 phosphorylation (pY705) was barely affected. NOD2-silencing had no significant effect on STAT1 or STAT3 phosphorylation.

Previous studies demonstrated that, upon IL-10 stimulation, STAT3 mainly mediates the anti-inflammatory functions of this cytokine, whereas signalling by STAT1 stimulates a pro-inflammatory response^33^. Therefore, we hypothesised that the reduction in IL-10-induced STAT1 phosphorylation observed in NOD1-silenced iDCs could shift the balance between STAT1 and STAT3 signalling towards STAT3. To analyse whether the IL-10/STAT3 axis is favoured in iDCs with reduced NOD1 levels, we monitored STAT3/DNA interactions upon IL-10 stimulation. To this end, we transfected iDCs with siRNA targeting NOD1 or a control siRNA, induced the cells with IL-10, and then performed a STAT3 DNA-binding assay. Our data show that NOD1-silencing resulted in reduced STAT1 phosphorylation (Fig. 3a,b), but also promoted binding of STAT3 to DNA (Fig. 3c). These data suggest that NOD1 depletion shifts IL-10-induced responses towards STAT3 activation, which might explain the increased *il10* and *march1* expression observed upon NOD1 silencing.

### SSH1 is involved in the regulation of STAT1 activity

**Figure 4:**
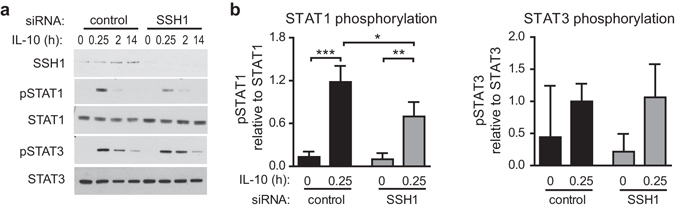
Depletion of SSH1 is correlated with diminished STAT1 activation. (**a/b**) Immature DCs were generated and transfected with a control siRNA or siRNA directed against SSH1. 72 hours post transfection, cells were stimulated with IL-10 (30 ng/ml) for the indicated time points and STAT activation was analysed by Western Blot. Tyrosine phosphorylation of STAT1 and STAT3 was detected by specific antibodies. To control for equal loading, total STAT protein was detected. One out of four independent experiments **(a)** and quantification of four experiments **(b)** are shown. Data represent mean and SD. For statistical analysis a one-way ANOVA with Tukey’s post hoc test was performed.

The fact that reduced NOD1 levels in iDCs dampen IL-10-induced STAT1 phosphorylation raised the question of how NOD1 mediates this negative effect on STAT1 activation. NOD1 has not been described to possess kinase or phosphatase activity. However, the phosphatase Slingshot Homolog 1 (SSH1) was recently identified as a novel NOD1-interaction partner^34^. The best-documented function of SSH1 is its ability to dephosphorylate cofilin, which results in cofilin activation and actin dynamics^35^. However, little is known about other functions of SSH1. To test whether SSH1 is able to modulate IL-10-induced STAT1 activation, we transfected iDCs with siRNA targeting SSH1 or a control siRNA and then examined IL-10-induced STAT1 and STAT3 activation. Silencing of SSH1 barely affected STAT3 phosphorylation, but resulted in a significant reduction of STAT1 phosphorylation upon IL-10 stimulation (Fig. 4a,b). Co-immunoprecipitation studies in HEK293T cells revealed no high-affinity interactions between endogenous STAT1 and NOD1 or SSH1 (Supplementary Fig. S4). This suggests that, although SSH1 and NOD1 both have the ability to modulate IL-10/STAT1 signalling, the effect is not likely mediated by direct interaction with STAT1.

### NOD1 and SSH1 silencing results in diminished *socs2* expression

**Figure 5:**
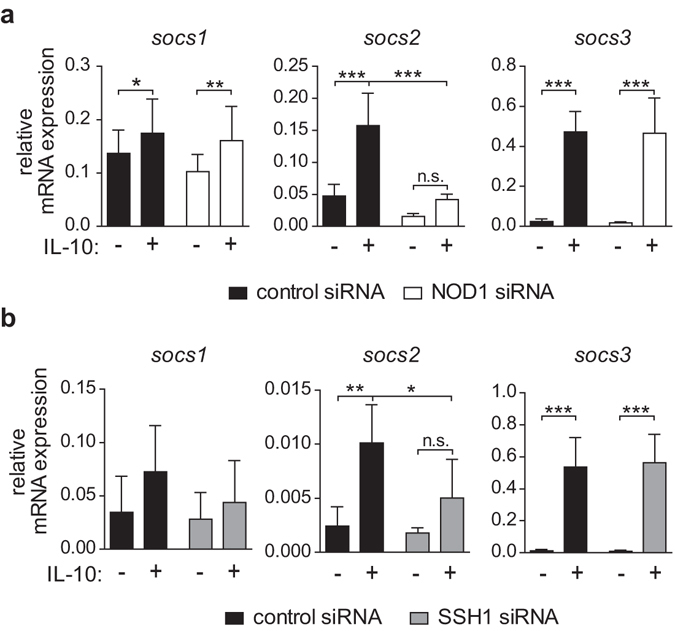
Silencing of NOD1 or SSH1 results in diminished SOCS2 mRNA expression. (**a/b**) iDCs transfected with a control siRNA or with siRNA directed against (**a**) NOD1 or (**b)** SSH1 were stimulated with IL-10 (30 ng/ml) for 2 hours, and *socs1*, *socs2* and *socs3* mRNA expression was analysed by q-RT-PCR. Data represent mean and SD of five independent experiments. For statistical analysis a one-way ANOVA with Tukey’s post hoc test was performed.

To further investigate the molecular mechanism resulting in reduced STAT1 phosphorylation upon NOD1 silencing, we examined whether suppressor of cytokine signalling (SOCS) proteins may be involved. This family of regulatory proteins preferentially inhibits cytokine-induced signalling pathways, with SOCS1 being the best-known and most potent suppressor of STAT1 activation^30, 36^. To analyse whether the decrease in STAT1 phosphorylation observed upon NOD1 or SSH1 silencing is associated with altered SOCS expression, we monitored *socs1*, *socs2* and *socs3* mRNA levels in NOD1- and SSH1-silenced cells. While IL-10 stimulation results in enhanced *socs1*, *socs2* and *socs3* expression in control cells, the ability of IL-10 to induce *socs2* mRNA is significantly impaired upon NOD1 and SSH1 silencing (Fig. 5a,b).

### SOCS2 augments IL-10-induced STAT1 phosphorylation

Our data show that NOD1 and SSH1 silencing results in decreased STAT1 activation (Figs 3 and 4) and reduced SOCS2 levels (Fig. 5). Moreover, we performed co-immunoprecipitation studies demonstrating an interaction between SOCS2 and NOD1, as well as between SOCS2 and SSH1 (Supplementary Fig. S5). This indicates that NOD1 and SSH1 might alter IL-10-induced STAT1 activation through modulation of SOCS2.

**Figure 6:**
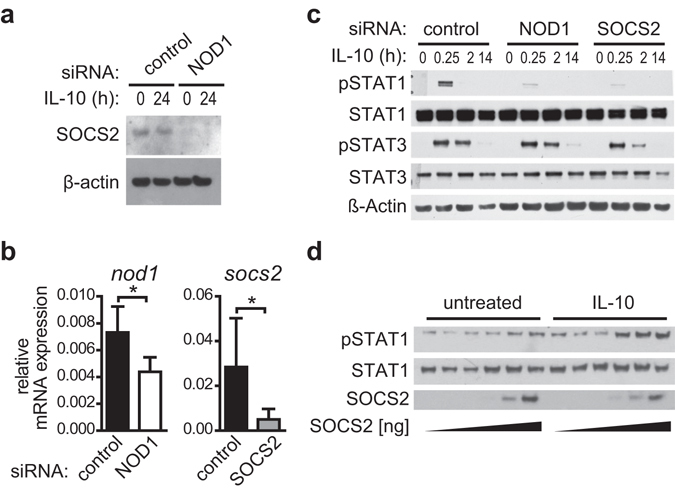
SOCS2 regulates IL-10-dependent STAT1 phosphorylation. (**a**) NOD1 silencing and control silencing were performed in iDCs. After 3 days of silencing, iDCs were induced with IL-10 (30 ng/ml) for 24 hours. SOCS2 and β-actin expression was monitored by Western Blot. One of two experiments is shown. (**b,c)** Immature DCs were transfected with a control siRNA or siRNA directed against NOD1 or SOCS2. (**b**) 72 hours post transfection, silencing efficiency was analysed by q-RT-PCR. Data represent mean and SD of three independent experiments. For statistical analysis a Student’s t-test was performed. **c** Transfected cells were stimulated with IL-10 (30 ng/ml) for the indicated time points to analyse STAT1 and STAT3 phosphorylation by Western Blot. To control for equal loading, total STAT protein and β-actin was detected. One representative experiment out of three is shown. (**d**) HEK293 cells were transiently transfected with expression vectors encoding SOCS2 and the IL-10 receptor subunits IL-10R1 and IL-10R2. 24 hours post transfection, cells were left untreated or stimulated with IL-10 (10 ng/ml) for 20 minutes. STAT1 phosphorylation and SOCS2 expression were analysed by Western Blot. To control for equal loading, total STAT1 was detected. One representative experiment out of three is shown.

Next we confirmed the decrease in SOCS2 expression upon NOD1 silencing at the protein level (Fig. 6a). To evaluate the role of SOCS2 in regulating STAT1 activity, we transfected iDCs with siRNA targeting SOCS2 or with a control siRNA, and monitored STAT1 and STAT3 activation by Western Blot. Similar to NOD1 depletion, SOCS2-silencing (Fig. 6b) resulted in decreased IL-10-dependent STAT1 phosphorylation. By contrast, STAT3 phosphorylation remained unaffected (Fig. 6c). In line with this, overexpression of SOCS2 in HEK293 cells led to enhanced STAT1 phosphorylation (Fig. 6d). These data show that SOCS2 does not act as negative feedback inhibitor in this case; instead, SOCS2 is positively correlated with IL-10-induced STAT1 phosphorylation.

### SOCS2 regulates SOCS1, and SOCS1 depletion specifically promotes IL-10 induced STAT1 activation

**Figure 7:**
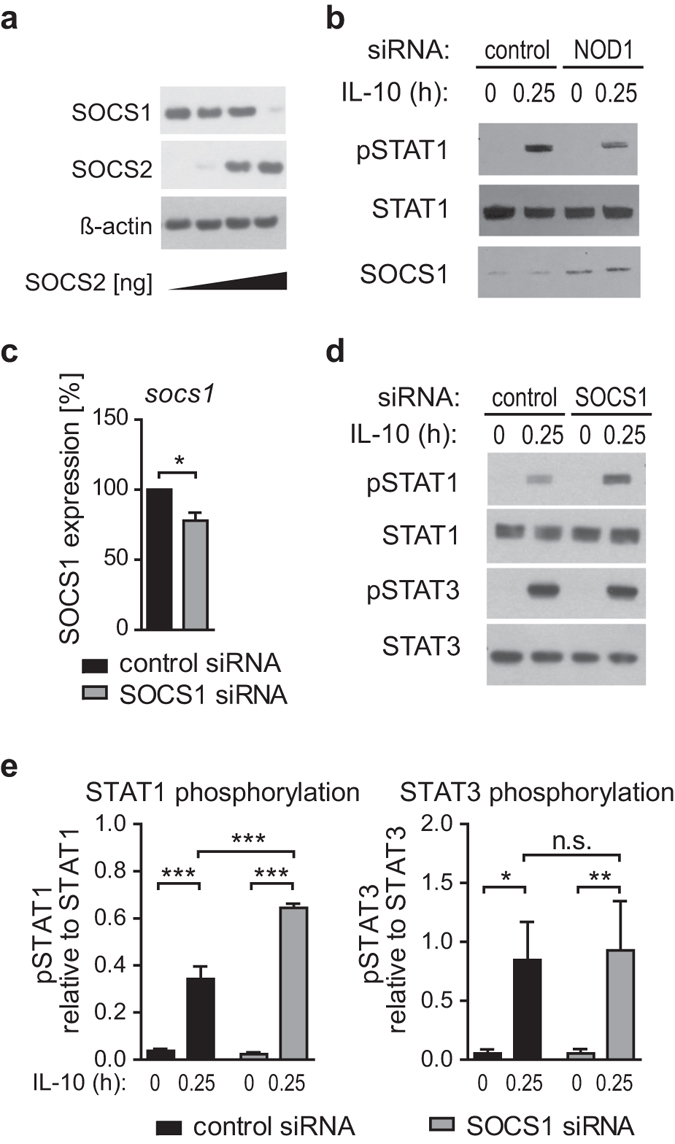
SOCS2 regulates SOCS1, and SOCS1 depletion specifically promotes STAT1 but not STAT3 activation. **(a**) Effect of overexpression of SOCS2 on SOCS1 levels. HEK293 cells were transfected with constant amounts of SOCS1 plasmid and increasing amounts of SOCS2 plasmid. 48 hours post transfection, protein levels of SOCS1 and SOCS2 were measured by Western Blot. One representative experiment out of three is shown. (**b**) NOD1 and control silencing of iDCs was performed for 72 hours. Thereafter, cells were induced with IL-10 (30 ng/ml) for 15 minutes and pSTAT1, STAT1 and SOCS1 levels were analysed by Western Blot. One of two experiments is shown. (**c,d,e**) Immature DCs were transfected with siRNA directed against SOCS1 or a control siRNA. **c** Efficiency of SOCS1 silencing in iDCs was analysed by q-RT-PCR. (**d**) 72 hours post transfection, cells were induced with IL-10 (30 ng/ml) for 15 minutes and STAT1/3 phosphorylation was monitored using Western Blot analysis. To control for equal loading, total STAT protein was detected. One out of three independent experiments is shown. (**e**) Values of three independent experiments were used for quantification. Data represent mean and SD. For statistical analysis a one-way ANOVA with Tukey’s post hoc test was performed.

SOCS2 was reported to target other SOCS family members for proteasomal degradation^37^. Amongst those, SOCS1 is the best-known feedback inhibitor of IFN-dependent STAT1 activation^38^ and was also reported to act as a negative regulator of IL-10 signalling^39^. To analyse whether SOCS2 may affect IL-10/STAT1 signalling by modulating SOCS1 protein levels, we first transfected HEK293 cells with an expression vector encoding SOCS1 along with rising concentrations of a vector encoding SOCS2. As shown in Fig. 7a, SOCS1 protein levels declined with increasing amounts of SOCS2.

In the context of this study, lower SOCS2 expression upon NOD1 silencing may enhance SOCS1 abundance in the cell. This assumption is supported by the observation that cells treated with NOD1 siRNA show enhanced SOCS1 protein expression compared to control cells (Fig. 7b). Moreover, IL-10-dependent STAT1 phosphorylation was again diminished upon NOD1 silencing. This suggests that elevated SOCS1 levels, as observed upon NOD1 silencing, may control STAT1 phosphorylation induced by IL-10. To demonstrate that SOCS1 is a key player in the specific suppression of the IL-10/STAT1 axis, we compared IL-10-induced activation of STAT1 and STAT3 in cells that have been transfected with siRNA targeting SOCS1 (Fig. 7c). Our data clearly show that silencing of SOCS1 results in an increase of STAT1 activation, whereas STAT3 was not affected (Fig. 7d,e).

Taken together, we demonstrate that NOD1 silencing suppresses IL-10-mediated STAT1 phosphorylation, whereas STAT3-DNA binding is enhanced. Moreover, NOD1 depletion results in reduced SOCS2 expression and increased SOCS1 protein levels, which may explain the specific inhibition of STAT1 phosphorylation. The alterations in IL-10-induced STAT1/STAT3 signalling caused by NOD1 silencing may thus allow for enhanced expression of IL-10 target genes and favour a more tolerogenic DC phenotype.

## Discussion

NOD1 has been established as an intracellular PRR that responds to bacterial peptidoglycan in the cytosol and induces the release of pro-inflammatory cytokines and chemokines^1, 2^. Several recent studies suggested that NOD1 is involved in further biological processes beyond pathogen recognition. For example, NOD1 provides a link between ER-stress-induced inflammation and innate immunity, is involved in tumourigenesis, senses the activity of Rho GTPase activity, and functions in cytoskeletal dynamics^7, 8, 10, 34^. All of those studies mainly focused on the ability of NOD1 to promote a pro-inflammatory immune response in terms of NF-κB activation and expression of pro-inflammatory cytokines. To identify a potential role of NOD1 in regulating anti-inflammatory responses, we analysed the effects of NOD1 on the IL-10-induced phenotype of human DCs. Here we showed that reduced NOD1 levels promote a more tolerogenic DC phenotype by decreasing IL-10-induced STAT1 activation via a mechanism that may involve the regulatory protein SOCS2.

We demonstrated that the reduction of STAT1 activation corresponds to enhanced STAT3 DNA-binding, which in turn correlates with increased IL-10 expression. In line with this, previous work reported that the pro-inflammatory cytokine IFN-γ is able to activate STAT1, which subsequently replaces STAT3 at the endogenous human *il10* promoter to dampen IL-10 production^40^. Moreover, in human macrophages, STAT3 is crucial for IL-10-induced expression of *il10* as well^28^. The fact that STAT1 counteracts STAT3 activation in other signalling pathways^41^, together with our observation that NOD1 silencing results in both reduced STAT1 phosphorylation and enhanced STAT3-DNA binding, indicates that STAT1 might counteract STAT3-DNA binding in the IL-10 signalling cascade. This idea is further supported by the finding that enhanced IL-10-induced STAT1 phosphorylation in monocytes reduces the suppressive capacity of IL-10 on mediators induced by lipopolysaccharide^42^, and it supports the hypothesis that the balance between STAT1-and STAT3-activation triggers the pro- or anti-inflammatory capacities of IL-10 signalling^33^.

As NOD1 is unlikely to possess kinase or phosphatase activity, we investigated a potential role of a novel NOD1 interaction partner, the phosphatase SSH1, in mediating the NOD1-dependent effects on IL-10 signalling. Originally described as a regulator of cofilin activation, SSH1 was recently shown to interact with NOD1 at F-actin structures. Furthermore, SSH1 deficiency has been linked to impaired NOD1 signalling upon *Shigella flexneri* infection^34^. Here we reported that SSH1 may be involved in modulating IL-10-induced STAT1 activation. However, SSH1 does not appear to be acting as a phosphatase on STAT1, because silencing of SSH1 resulted in lower IL-10-induced STAT1 phosphorylation.

SOCS2 is a known inhibitor of growth hormone-induced STAT5 signalling and was also shown to act as a feedback inhibitor of TLR-induced DC activation^43, 44^. Whereas IL-10-induced STAT activation is regulated by SOCS1 and SOCS3, SOCS2 does not affect this pathway in Ba/F3 cells^39^. In accordance, we demonstrated that SOCS2 does not alter IL-10-induced STAT3 phosphorylation in iDCs. However, the observed correlation between SOCS2 deficiency and decreased IL-10-induced STAT1 activity might be explained by our finding that increasing SOCS2 expression correlates with decreasing SOCS1 protein levels. Whereas SOCS1 and SOCS3 each possess a kinase inhibitory region (KIR) to inhibit the activity of JAKs, SOCS2 lacks this domain. Thus, the inhibitory function of SOCS2 relies on competitive binding via its SH2-domain and, even more important, on linking an interacting protein to the ubiquitination machinery and targeting it for proteasomal degradation^45^. Enhanced SOCS1 degradation resulting from the direct interaction of SOCS1 and SOCS2 was shown by Wu and colleagues^46^. Accordingly, it was reported that SOCS2 promotes SOCS1 degradation in epithelial cells^47^. Our data indicate a correlation between diminished NOD1 expression and a decrease in SOCS2 expression but an increase in SOCS1 levels. Therefore we propose that SOCS2 mediates downregulation of IL-10-induced STAT1 phosphorylation by regulating SOCS1 levels. Moreover, our results reveal that SOCS1 specifically modulates IL-10-induced STAT1, but not STAT3, in iDCs. This finding is supported by results from SOCS1-deficient macrophages, showing specifically increased IL-10-induced STAT1, but not STAT3, phosphorylation^48^.

With respect to the DC phenotype, our results imply that NOD1 silencing not only modulates the IL-10-signalling cascade, but also promotes a tolerogenic DC phenotype. NOD1-silenced DCs express higher levels of *il10* and *march1* and significantly down-regulate both expression of the co-stimulatory molecule CD86 and secretion of IL-12. Moreover, NOD1-silenced DCs are more potent to prime CD25^+^FOXP3^+^ T cells. Interestingly, a similar phenotype, characterised by diminished expression of co-stimulatory molecules, has been observed in human DCs infected with live *Helicobacter pylori* ^49^. Moreover, *H. pylori* was shown to induce IL-10 secretion in DCs, which potentially promotes chronic infection and may also cause gastric cancer^50^. In addition to IL-10, NOD1, SOCS1 and SOCS2 have all been implicated in the context of gastrointestinal malignancies. In those studies, low NOD1 levels as well as increased IL-10 expression were detected in biopsies of gastric cancer tissue compared to healthy tissue^10, 51^. Moreover, SOCS1 was described as an oncogene in colorectal cancer, whereas SOCS2 is downregulated in this disease^52, 53^. Although the present study gives important new insights into the potential crosstalk between NOD1, IL-10-induced STAT1-STAT3 signalling and SOCS protein functions in haematopoietic cells, potential relationships amongst these proteins in the context of cancer need to be further investigated.

Taken together, this study provides additional evidence for peptidoglycan-independent functions of NOD1 and suggests that NOD1 has the ability to suppress the tolerogenic capacities of IL-10-stimulated DCs. Thus, NOD1 not only contributes to pro-inflammatory immune responses induced upon ligand binding, but may also indirectly promote inflammation by suppressing anti-inflammatory signalling pathways.

## Methods

All studies involving human cells were conducted in accordance with the guidelines of the World Medical Association’s Declaration of Helsinki. In this study, we used immune cells from human buffy coats. Our national regulations do not require informed consent in the case of anonymous blood cells discarded after plasmapheresis (buffy coats), therefore no additional approval by the local ethics committee is required. According to university guidelines, the project was approved by the head of the Department of Molecular Biology, University of Salzburg, Austria.

### Generation of monocyte-derived immature dendritic cells (iDCs)

Immature DCs were generated from buffy coats from healthy, anonymous donors (provided by the blood bank Salzburg, Austria) as described previously^43^. Briefly, density gradient centrifugation using Histopaque-1077 (Sigma) was performed to isolate PBMCs. ACK buffer (150 mM ammonium chloride, 10 mM potassium bicarbonate, 0.1 mM EDTA) was used to lyse erythrocytes, and monocyte adherence was performed for 75 min at 37 °C and 5% CO_2_ in DC medium [RPMI 1640 (Sigma), 10% foetal calf serum (PAA), 2 mM l-glutamine, 100 U/ml penicillin/streptomycin (Sigma), 50 μM β-mercaptoethanol (Gibco Laboratories)]. After washing, adherent monocytes were cultured in DC medium supplemented with 50 ng/ml GM-CSF and 50 ng/ml IL-4 (generous gift from Novartis) for 7 days to generate immature iDCs or with additional 30 ng/ml IL-10 (R&D Systems) to obtain tolerogenic DCs. On day 3 of differentiation, fresh supplemented medium was added to the cells.

### siRNA-based silencing in human iDCs

For silencing experiments, iDCs were transfected with small interfering RNAs directed against SOCS1 (HSS144821), SOCS2 (VHS41173) (Life Technologies), CARD4/NOD1 (SI00084483), CARD15/NOD2 (SI00133049), SSH1 (SI00123585) or Allstars negative Control (Qiagen) on day 7 of differentiation. Transfection was performed with Lipofectamine RNAiMax reagent (Life Technologies) according to the manufacturer’s guidelines. Briefly, 2 × 10^5^ iDCs were plated in 100 µl fresh DC medium per well and transfected with 100 pmol of siRNA and 1.5 µl Lipofectamine RNAiMax in 100 µl Opti-MEM (Life Technologies). After incubation for 6 h, 800 µl of fresh DC medium were added and the cells were incubated for another 3 days before they were used for further experiments. Silencing efficiency was analysed by qRT-PCR or Western Blot analysis.

### DC phenotyping by flow cytometry and enzyme-linked immunosorbent assay (ELISA)

Surface expression of DC activation markers was analysed using a FACS Canto II flow cytometer (BD Biosciences) and the following antibodies: CD86-PE (IT2.2), HLA-DR-APC-Cy7 (L243), PD-L1-PE-Cy7 (MIH1), PD-L2-APC (MIH18) obtained from BD Biosciences, ILT2-APC (HP-F1), ILT3-PE (ZM4.1) and ILT4-FITC (27D6) obtained from eBioscience. The median fluorescence intensity of DCs was compared.

IL-10 as well as IL-12 secretion was monitored by ELISA, both obtained from Peprotech, and performed according to manufacturer’s instructions.

### Isolation of naïve T cells and co-culture experiments

Naïve CD4^+^ T cells were isolated from PBMCs using the untouched Human Naïve CD4^+^ T cell isolation kit II (Miltenyi Biotec), according to the manufacturer’s recommendations. After isolation, T cells were co-cultured with immature iDCs or tolerogenic DCs at a ratio of 10:1 in IMDM containing 5% FCS (PAA) and 100 U/ml penicillin/streptomycin. After 7 days, 20 U/ml IL-2 were added. On day 10 of co-culture, T cells were stimulated with 10 µg/ml plate-bound α-CD3 (clone OKT3, eBioscience) and 1 µg/ml PMA (Sigma). 48 h post-stimulation, cytokine secretion was analysed by Multiplex Assay (ProcartaPlex Human Th1/Th2/Th9/Th17/Th22/Treg Cytokine Panel 18 plex, e-Bioscience), and FOXP3 and CD25 (eBioscience) expression was monitored by flow cytometry. CD25^+^, FOXP3^+^ T cells were gated on CD3^+^, CD4^+^, live cells after doublet exclusion (Supplementary Fig. S6).

### Characterisation of T cells by flow cytometry

To analyse the number of regulatory T-cells after co-culture with iDCs or tolerogenic DCs, live CD4^+^ T cells were analysed for CD25 and FOXP3 expression using CD25-APCeFluor780, FOXP3-e450 (e-Bioscience), Fixable Viability Dye eFluor® 520 (Affymetrix) and CD4-BD-Horizon V500 (BD Biosciences). The percentage of CD25^+^/FOXP3^+^ positive cells in each sample was measured by using a FACS Canto II flow cytometer (BD Biosciences) and analysed using Flow Jo software.

### Multiplex immunoassay

Supernatants of re-stimulated T cells were analysed for cytokine expression with a ProcartaPlex Human Th1/Th2/Th9/Th17/Th22/Treg Cytokine Panel (18 plex, e-Bioscience). Briefly, equal amounts of Bead Mix B and C were mixed and washed twice with wash buffer before they were added to the standard samples or supernatants in a 96-well V-bottom plate and left overnight at 4 °C on an orbital shaker. The next day, equal amounts of Antibody solutions B and C were diluted in Assay Buffer and washed 3 times, before they were added to the samples. After 30 min, wash buffer was added and the plate was washed 3 times by centrifugation at 1500 × g for 4 min and discarding the supernatant. Streptavidin-PE solution was diluted in Assay Buffer 1:2 and added to the cells for another 30 min. Prior to analysis the plate was washed again 3 times and beads were resuspended in 100 µl drive fluid after the last washing step. Recording was performed on a Luminex MagPix (Luminex) and data analysis was done using Procarta Plex Analyst Software (e-Bioscience).

### RNA isolation and quantitative real-time PCR

Total RNA was isolated from 2 × 10^5^ cells by using TRI Reagent (Sigma) and reverse-transcribed with RevertAid H Minus M-MulV reverse transcriptase (Thermo Scientific) according to the manufacturer’s instructions. RNA expression was quantified by performing quantitative real-time PCR (q-RT-PCR) on a Rotorgene 3000 (Corbett Research) with iQ SYBR Green Supermix (Bio-Rad) and the primers listed below. The relative mRNA content (x) was calculated using the formula x = 2^−Δct^, where Δct describes the difference between the threshold cycles (ct) of the gene in question and the reference gene large ribosomal protein P0 (RPLP0). The specificity of the PCRs was monitored by recording a melting curve for the PCR products. The following primer pairs were used for detection: NOD1: sense 5′-GTCATGCTAGAAGAACTCTGCCTGGAGGAGA-3′, antisense 5′-CAGCATCCAGATGAACGTG-3′^54^, NOD2: sense 5′-GTCATGCTAGAAGAACTCTGCCTGGAGGAGA-3′, antisense 5′-GCCCCTAGGTAGGTGATGCAGTTATTGGAC-3′^54^, IL-10: sense 5′-AGGGCACCCAGTCTGAGAACA-3′, antisense 5′-CGGCCTTGCTCTTGTTTTCAC-3′^43^, SOCS1: sense 5′-TTGGAGGGAGCGGATGGGTGTAG-3′, antisense 5′-AGAGGTAGGAGGTGCGAGTTCAGGTC-3′^43^, SOCS2: sense 5′-CCAAATCAACCAAAAAAAGTGACCATGAAGTCCTG-3′, antisense 5′-CGGGGATTGAGTTGCACCTGTATAGCATGATATTC-3′^43^, SOCS3: sense 5′-ATACTATACCTTCCTGTACCTGGGTGGATGGAGCG-3′, antisense 5′-TGAGTATGTGGCTTTCCTATGCTGGGTCCCTCT-3′^43^, MARCH1: sense 5′-TCCCAGACAGACGACCTCATGGTTTT-3′, antisense 5′-AAAATCTTCCCACCTCAGCCCCAG-3′, RPLP0: sense 5′-GGC ACC ATT GAA ATC CTG AGT GAT GTG-3′, antisense 5′-TTG CGG ACA CCC TCC AGG AAG-3′^43^.

### Western blotting and SDS-PAGE

After induction with IL-10 (30 ng/ml) for the indicated time points, 2 × 10^5^ cells were lysed in 80 µl of 2x SDS sample buffer (Bio-Rad), separated on a precast NuPAGE 4–12% gradient gel (Life Technologies) and transferred onto a nitrocellulose membrane (Bio-Rad). Blocking was performed for 1 h in TBS containing 0.1% Tween 20 and 5% nonfat dry milk. Antibodies against p-STAT1-Tyr701 (Cat no. 9167), STAT1 (Cat no. 9172), p-STAT3-Tyr705 (Cat no. 9145), STAT3 (Cat no. 4904), β-Actin (Cat no. 4970), SOCS1 (Cat nos 3957 and 3950), SOCS2 (Cat no. 2779), SSH1 (Cat no. 13578) and HRP-linked anti-rabbit secondary antibody (Cat no. 7074) were all purchased from Cell Signaling Technology and used according to the manufacturer’s instructions. West Pico PLUS Chemiluminescent Substrate (Thermo Fisher) and BioMax film (Kodak) were used for detection. ImageJ (NIH) was used for quantification of Western Blots.

### Transfection of HEK293 cells – effect of SOCS2 on STAT1 activation

HEK293 cells were cultured in DMEM medium (Sigma) supplemented with 10% FCS, 2 mM L-glutamine and 2 mM MEM non-essential amino acids and plated at a density of 1.2 × 10^5^ cells/ml. 24 h after seeding, the cells were transfected with expression vectors for pcDNA3.1-SOCS2^55^, pORF9-hIL-10R1 and pORF9-hIL-10R2 (InvivoGen) and pCDNA3.1^+^ using Lipofectamine 2000 (both Life Technologies) according to the manufacturer’s guidelines. 3 ng of IL-10R1 and 3 ng IL-10R2 (6 ng in total) were used and SOCS2 was added in the following ratios (IL-10R:SOCS2): 1:0; 1:0.1; 1:0.5; 1:2.5; 1:12.5; 1:62.5. To ensure that equal amounts of plasmids were used for transfection in each sample, appropriate amounts of pcDNA were added to a total of 381 ng of plasmid per well. 24 h post transfection, the cells were left untreated or stimulated with 3 ng/ml IL-10 (R&D Systems) for 20 min. Cells were lysed in 2x SDS Buffer (Bio-Rad) and analysed by SDS-PAGE and Western Blot.

### Transfection of HEK293 cells – crosstalk between SOCS1 and SOCS2

1.2 × 10^5^ HEK293 cells/ml were seeded and incubated for 24 h then transfected with 75 ng of a vector encoding SOCS1 (pcDNA3.1-SOCS1) ^55^ together with 0, 15, 75 or 375 ng of a plasmid encoding SOCS2 (pcDNA3.1-SOCS2)^55^. To ensure that equal amounts of total DNA were transfected per well, appropriate amounts of the empty vector (pcDNA3.1) were added. 48 h post transfection, the cells were lysed in 2x SDS Buffer and used for analysis.

### Nuclear extracts and STAT3 binding assay

Nuclear extracts were prepared using the nuclear extract kit purchased from Active Motif according to the manufacturer’s guidelines. Briefly, 8 × 10^5^ cells were lysed in 250 µl hypotonic buffer. After centrifugation the supernatant contains the cytosolic fraction of protein, which was analysed for total STAT3 protein by Western Blot. The pellet was re-suspended in 25 µl of complete lysis buffer and vortexed for 10 seconds. After incubation for 30 min and centrifugation, the supernatant contains the nuclear proteins. The STAT binding assay (Active Motif) was performed according to the manufacturer’s instructions. Briefly, 30 µl binding buffer and 20 µl of nuclear extract per well were incubated for 1 h before 100 µl of STAT3 antibody solution was added. After 1 h incubation, the wells were washed 3 times and. 100 µl HRP-conjugated antibody was added. After extensive washing, developing solution was added and incubated for a short time before stop solution was used to prevent overdeveloping. Absorbance was measured at 450 nm with a reference wavelength of 650 nm. STAT3 binding relative to total STAT3 amount in the cytosol was calculated.

### Statistics

GraphPad Prism 5 software was used for statistical analysis. Data are expressed as mean plus standard deviation (SD). P values between two groups were calculated using a two-tailed Student’s *t* – test. To compare multiple groups for statistical differences a one-way ANOVA with Tukey’s post-hoc test was performed. P values of p < 0.05 were considered statistically significant. (*p < 0.05, **p < 0.01, ***p < 0.001, n.s. not significant).

## Electronic supplementary material

MOESM1_ESM.
