# Supplementary material for: NOD1 modulates IL-10 signalling in human dendritic cells
Source: Sci Rep. 2017 Apr 21;7:1005. doi: 10.1038/s41598-017-00691-x (PMC5430717; doi:10.1038/s41598-017-00691-x)
Supplement: Supplementary file 1 — MOESM1_ESM. [file 41598_2017_691_MOESM1_ESM.doc]

**NOD1 modulates IL-10 signalling in human dendritic cells**

**Authors**

**Theresa Neuper^1^, Kornelia Ellwanger^2^, Harald Schwarz^1^, Thomas A. Kufer^2^, Albert Duschl^1^ and Jutta Horejs-Hoeck^1^***

**1 Department of Molecular Biology, University of Salzburg, Salzburg, Austria**

**2 Institute of Nutritional Medicine, Department of Immunology, University of Hohenheim, Stuttgart, Germany**

**Supplementary Methods**

Activation of monocyte-derived immature dendritic cells by PRR ligands and IL-10

Immature DCs were generated as described previously ^1^ and stimulated with 30 ng/ml IL-10 (R&D Systems), 10 µg/ml iE-DAP, 10 µg/ml MDP and 10 µg/ml Poly I:C.

Quantitative real-time PCR

The following primer pairs were used for detection:

IL-10: sense 5'-AGGGCACCCAGTCTGAGAACA-3', antisense 5'-CGGCCTTGCTCTTGTTTTCAC-3'^1^, MARCH1: sense 5'TCCCAGACAGACGACCTCATGGTTTT-3', antisense 5'-AAAATCTTCCCACCTCAGCCCCAG- 3', IFNα2: sense 5'-gtgctcagctgcaagtcaag-3', antisense 5'-tctgctggatcatctcatgg-3', TNFα: sense 5'-caagcctgtagcccatgttg-3', antisense 5'-gaggttgaccttggtctggta-3', NOD1: sense 5'-GTCATGCTAGAAGAACTCTGCCTGGAGGAGA-3', antisense 5'-CAGCATCCAGATGAACGTG-3' ^2^, RPLP0: sense 5'- GGC ACC ATT GAA ATC CTG AGT GAT GTG-3', antisense 5'-TTG CGG ACA CCC TCC AGG AAG-3'^1^.

Comparison of different NOD1 siRNAs

For silencing experiments, iDCs were transfected with small interfering RNAs directed against CARD4/NOD1 (#3: SI00084483, #2: SI00084462, #1: SI00084476) or Allstars negative Control (Qiagen) on day 7 of differentiation.

Immunoprecipitation

For immunoprecipitation, HEK293T cells were transiently transfected with plasmids coding for FLAG-tagged NOD1 or FLAG-tagged-SSH1 and SOCS2 (3.5 µg total plasmid DNA per 6-cm dish) using Lipofectamine 2000 (Invitrogen) according to the manufacturer's conditions, and incubated for 48 h. Cells were lysed in NP-40 buffer (150 mM NaCl, 0,5% NP-40, 50 mM Tris-HCl, pH 7.4) containing phosphatase inhibitors (20 mM ß-glycerophosphate, 5 mM NaF, 100 µM Na_3_VO_4_, and Complete protease inhibitor cocktail [Roche]). Lysates were cleared for 20 min at 14,000 g at 4°C. Immunoprecipitation was subsequently carried out by incubating cell extracts with anti-FLAG beads (M2 agarose resin; Sigma-Aldrich) for 4 h at 4°C. Beads were precipitated by centrifugation and washed five times in NP-40 buffer before sodium dodecyl sulphate loading buffer was added. Typically, about 10 to 20 times more precipitate than input was loaded into the gel. Proteins were separated by SDS-PAGE and transferred by semidry Western transfer to a nitrocellulose membrane (Bio-Rad). Proteins were detected by successive incubation of the membrane with primary and secondary antibodies and by a final incubation with SuperSignal West Femto maximum sensitivity substrate (Pierce).

**Supplementary Figures S1-S6**


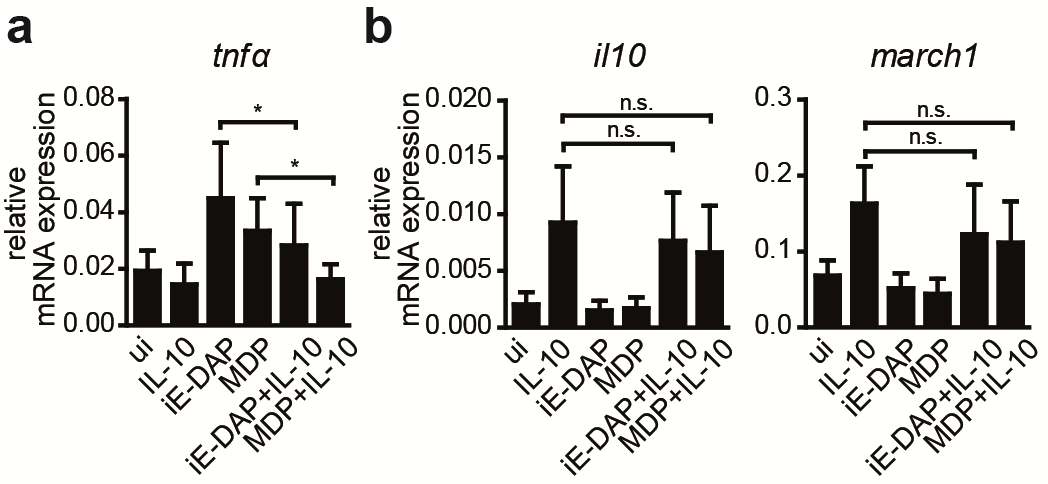


**Supplementary Figure S1 NOD1 and NOD2 activation by iE-DAP and MDP does not reduce IL-10 target gene expression.** Human iDCs were left untreated or stimulated with IL-10 (30 ng/ml), iE-DAP (10 µg/ml), MDP (10 µg/ml) or the indicated combinations. **a** To control for NOD1 and NOD2 activation, *tnf*α expression is shown. **b**  Two hours post induction, mRNA expression of IL-10 target genes (*il10, march1*) was monitored. Data represent mean and SD of five independent experiments. For statistical analysis a one-way ANOVA with Tukey’s post hoc test was performed.


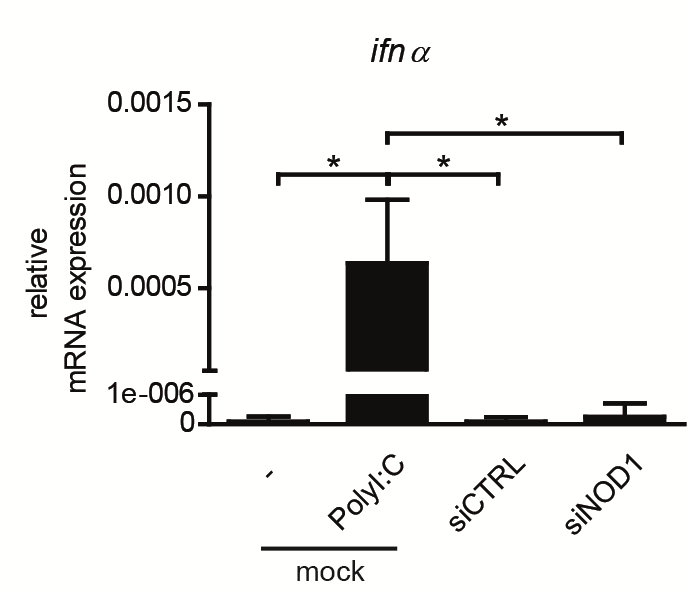


**Supplementary Figure S2 IFNα response upon silencing.** Human iDCs were transfected with siRNA directed against NOD1 or a control siRNA, or treated with the transfection reagents without siRNA (mock). Mock-treated cells were either left untreated or incubated with Poly:IC (10 µg/mL) for 6 hours. 72 hours post transfection, ifnα expression was analysed by q-RT-PCR; ifnγ expression was below the detection range. Data represent mean and SD of three independent experiments. For statistical analysis a one-way ANOVA with Tukey’s post hoc test was performed.


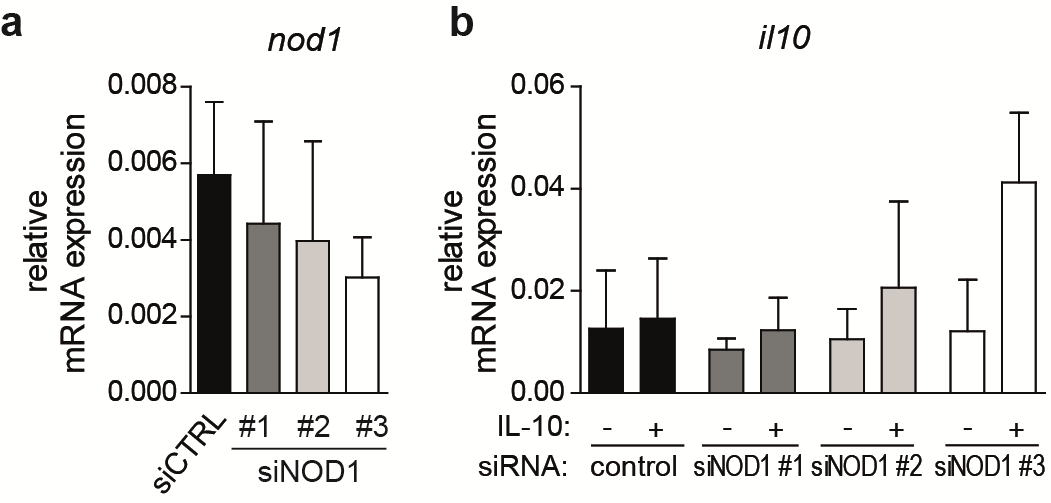


**Supplementary Figure S3 Comparison of 3 NOD1 siRNAs. A** Silencing efficiency of three siRNAs targeting NOD1 was compared by analysing NOD1 mRNA expression 72 hours post transfection. Data represent mean and SD of three independent experiments. **B** Cells silenced with three different siRNAs were treated with 30 ng/ml IL-10 for 2 hours and IL-10 mRNA expression was measured. Data represent mean and SD of three independent experiments.


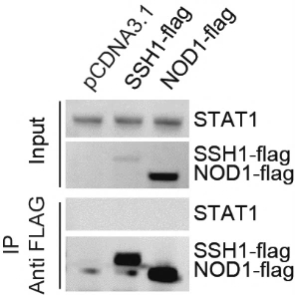


**Supplementary Figure S4 Co-immunoprecipitation assay reveals no high-affinity interaction between STAT1 and NOD1 or SSH1.** Immunoprecipitation of ectopically expressed FLAG-tagged NOD1 or FLAG-tagged SSH1 in HEK293T cells. FLAG-NOD1 and FLAG-SSH1 were precipitated using anti-FLAG antibody, and immunoprecipitates were probed for the presence of endogenous STAT1.


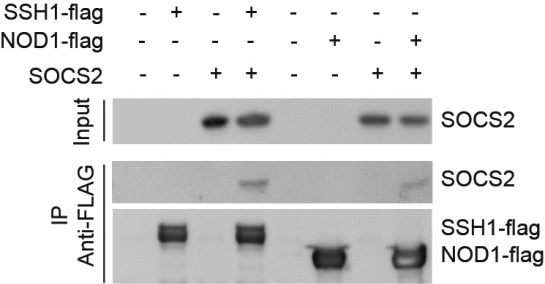


**Supplementary Figure S5 NOD1 and SSH1 interact with SOCS2.** Plasmids encoding FLAG-tagged NOD1 and FLAG-tagged SSH1 were overexpressed in combination with a plasmid encoding SOCS2. NOD1 and SSH1 were precipitated using an anti-FLAG antibody and the immunoprecipitates were probed for the presence of ectopically expressed SOCS2.


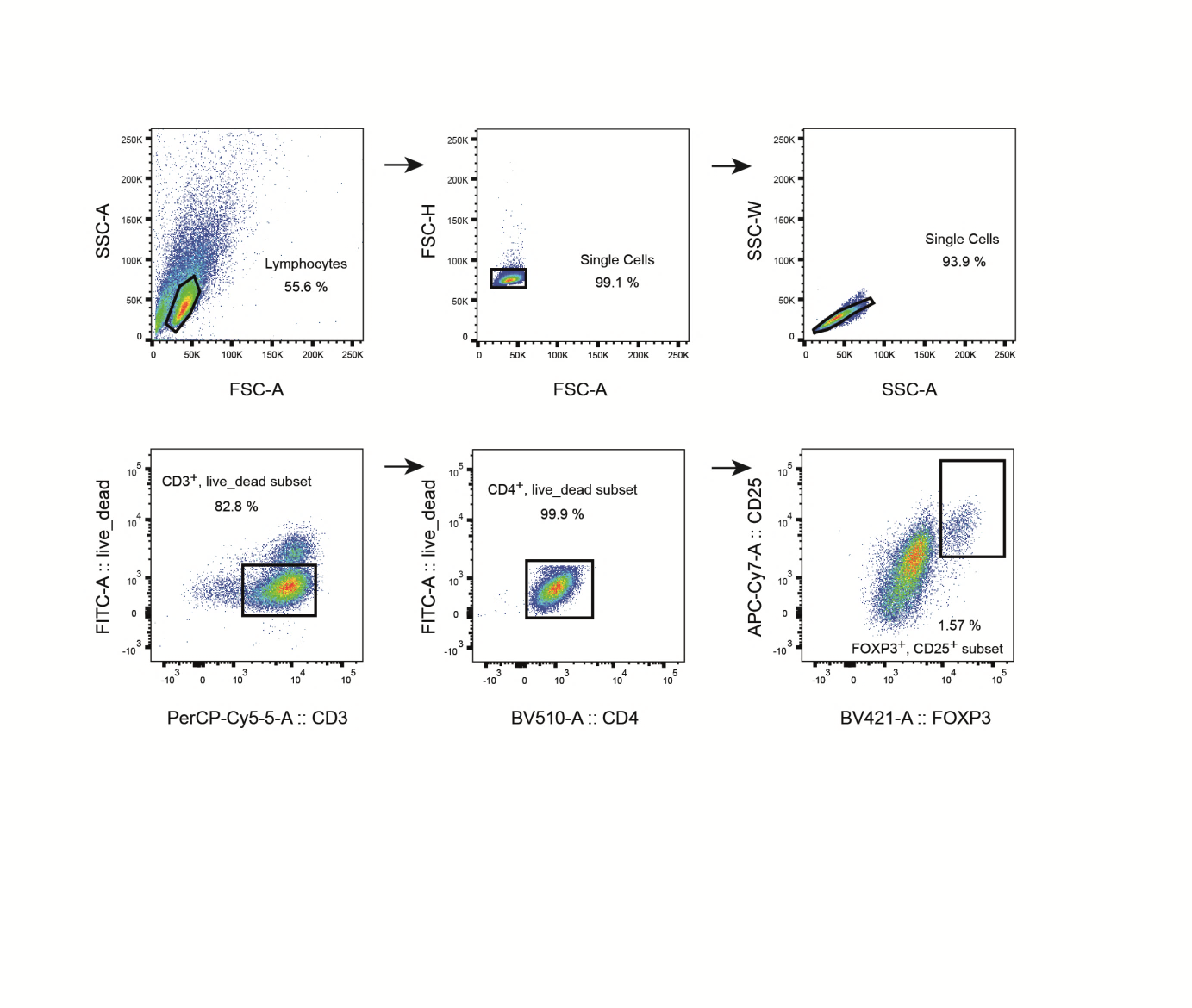


**Supplementary Figure S6 Gating strategy for T cells.** CD25^+^, FOXP3^+^ T cells were gated on CD3^+^, CD4^+^, live cells after doublet exclusion.

**References**

1. Posselt G, Schwarz H, Duschl A, Horejs-Hoeck J. Suppressor of cytokine signaling 2 is a feedback inhibitor of TLR-induced activation in human monocyte-derived dendritic cells. *J Immunol* **187**, 2875-2884 (2011).

2. Bielig H*, et al.* NOD-like receptor activation by outer membrane vesicles from Vibrio cholerae non-O1 non-O139 strains is modulated by the quorum-sensing regulator HapR. *Infection and immunity* **79**, 1418-1427 (2011).
